# A protocol for the establishment and evaluation of an older adult stakeholder panel for health services research

**DOI:** 10.12688/hrbopenres.12979.2

**Published:** 2020-07-20

**Authors:** Mairéad Conneely, Pauline Boland, Aoife O'Neill, David Byrne, Sinéad Cronin, Dominic Quinn, Dominic Trépel, Siobhán Leahy, Jon Salsberg, Rose Galvin, Katie Robinson

**Affiliations:** 1 School of Allied Health, Faculty of Education and Health Sciences, Ageing Research Centre, Health Research Institute, University of Limerick, Limerick, Ireland, V94 T9PX, Ireland; 2 HRB Centre for Primary Care Research, Mercer Building, Royal College of Surgeons in Ireland, Dublin 2, Ireland, DO2 YN72, Ireland; 3 Trinity Institute of Neurosciences, School of Medicine, Trinity College Dublin, Dublin 2, Ireland, DO2 PN40, Ireland; 4 Graduate Entry Medical School Ireland, University of Limerick, Limerick, Ireland, V94 T9PX, Ireland

**Keywords:** Older adults, public and patient involvement, research participation, collaboration, engagement.

## Abstract

**Background:**

There has been a policy shift towards public and patient involvement (PPI) in population health and health services research in Ireland and internationally. Despite growing evidence that PPI can have positive impacts on the quality and appropriateness of health research, little is known about the involvement and impact of older adults as research partners. The aim of this study is to 1) describe the process of establishing a PPI panel of older adults, family carers and ageing research academics and 2) to evaluate the impact of this research partnership on all research partners.

**Methods:**

A partnership-focused framework will guide the recruitment and establishment of a PPI panel of older adults, family carers and academic researchers. Eight to ten older adults and four to six family carers with experience of using health services will be recruited through gatekeepers in a range of non-governmental, voluntary, and community organisations in the Mid-West region of Ireland. Academic researchers will be recruited through the Ageing Research Centre at the University of Limerick. To evaluating the impact of the research partnership on all members of the PPI panel we propose to record an activity log, maintain a record of all meeting, panel discussions and conduct individual interviews with all members of the research team at key time points. The final plan for evaluation will be negotiated and agreed with all members of the PPI panel. Data will be transcribed, managed in NVivo and analysed using an inductive approach to thematic analysis. Dissemination of research findings will be facilitated by the research partnership team of academics and older adults.

**Discussion:**

This study will identify learning about the process of establishing a PPI panel guided by a partnership-focused framework and will evaluate the impact of participation in a PPI panel for all members of the research team.

## Introduction

By 2031, it is forecasted that there will be more than one million older adults (aged 65 years and older) in Ireland, representing an increase from 13% (2015) to 20% of our overall population ^1^. Nationally, the Health Services Executive (HSE) Planning for Health Report (2017) predicts a significant increase in healthcare service use over the next decade ^1^.Health services need to respond to these changing demographics, as older adults are major service users of hospital based acute care ^2, 3^ and community based models of care. Furthermore, to enhance healthcare of older adults there have been calls to focus on patient experience and patient preference ^4^, ^5^.

It is widely acknowledged that patients and the public should be involved as research partners to identify meaningful research priorities and to enhance the design and implementation of health services that are responsive to the changing needs and preferences of service users ^2^. The term “Public and Patient Involvement” (PPI) is described by the United Kingdom’s National Institute for Health Research body INVOLVE as “an active partnership between patients and the public and researchers ^6^. The research is “being carried out ‘with’ or ‘by’ members of the public”, not just ‘to’, ‘about’ or ‘for’ them” ^6^. There are many terms used in the literature to describe “involvement” of lay users of health services ^7– 9^. These terms include community members, stakeholders, public, citizens, consumers of health-care services, patient and service users ^10– 12^. In this paper, our preferred term is “PPI panel of older adults”.

In recent years, there has been an increased drive to involve patients and the public in health research including moral, practical and pragmatic arguments ^13– 15^. Morally, patient involvement is recommended on the grounds that people affected by a condition have a right in the decision process about research that may affect them. From a practical perspective, involving patients in research is intended to increase the relevance ^13^, accountability and transparency of research ^16^. Patient involvement brings a lived-experience perspective to research, with the potential to extend the range of people represented in research and improve dissemination of key findings beyond academic audiences ^15– 18^. From a research funding perspective, the British National Institute of Health recognised that public and patients can shape important aspects of health care research and established the INVOLVE organisation in 1996. Similarly, the Canadian Institutes for Health Research developed a Strategy for Patient Outcome Research; and the Patient-Centred Outcomes Research Unit was established in the United States in 2010 ^15^. The Health Research Board (HRB), an Irish state agency that supports research to improve health and transform patient care, recognised the importance of PPI in its 2016–2020 strategy ^19^. Inclusion of PPI is now a standard feature of most HRB funding calls and the HRB PPI Ignite Awards 2017 focused on promoting institutional-wide PPI within Irish universities ^20^.

Though most researchers, funders and patient advocacy groups agree on the merit and importance of PPI, the impact of PPI is not readily measurable. A Cochrane review, exploring the effects of patient involvement in developing healthcare research, concluded that involving patients in the research process may lead to an output that is more readable and understandable by other patients ^21^. The authors reported that involvement with patients was feasible in most of the randomised controlled trials included in the review. A further review including 66 studies examining the impact of PPI in health and social care research reported that PPI enhanced the quality of research, ensuring relevance and appropriateness ^11^. The authors reported that PPI may have a more positive impact when PPI is embedded throughout the study and when patients and the public are involved as partners in the research team. Many studies within this systematic review reported positive impacts in the initial stages of research, with users developing user-friendly information, user-focused research objectives and more appropriate recruitment strategies. The results of this review also reported that user involvement may achieve better dissemination of research findings due to the influence of research-users in the communities for whom the findings are intended. PPI has also been incorporated into data analysis ^22– 24^ with positive reports of this involvement in studies with older adults ^22^. A major impetus for evaluating PPI is to build evidence that involvement has a positive impact on research processes and outcomes ^25^

There are many documented challenges to embedding PPI in the research cycle ^15, 26^. Challenges to PPI are reported where public and patients are involved sporadically in the study with no clear defined role ^9^. There has also been concern that involvement was tokenistic ^27^. Other challenges reported in the literature include patient frustration with the research process which involved training, attendance at meetings and power issues between researcher and PPI contributors ^13^. Poor quality of reporting of PPI has been cited as one of the barriers to understanding the impact of PPI in research ^17^.

Evidence for evaluating the involvement and impact of older adults in health research is sparse. A systematic review in 2007 analysed 30 studies which involved older adults in commissioning, prioritising, designing or disseminating research. Involvement varied from completing questionnaires or taking part in an interview ^10^. The review found only four studies which formally evaluated involving older people in the research studies using different evaluation methods ranging from questionnaire to an ethnographic approach. A recent systematic review evaluated the impacts of involving older adults in health and social care research on older co-researchers, academic researchers and research processes ^28^. The review analysed nine articles using a qualitative methodology evaluating older adults' involvement in research. The authors reported that all nine articles described beneficial impacts for older co-researchers in terms of social benefits, new learning, being valued, developing relationships with academics and shared workloads. Five studies in the review reported beneficial impacts for academic researchers which included learning from the experiences of the co-researchers during recruitment, data collection and dissemination. The challenges reported for older co-researchers were reported in seven articles detailing issues with a demanding workload and dissatisfaction with the level of involvement. Whilst challenges were reported with all studies reporting a lengthy participatory process, the authors concluded that the benefits outweighed the challenges.

Patient involvement in healthcare research is feasible and adaptable to many contexts and settings ^15, 29^. The study aims to:

- Describe the process of establishing a PPI panel of older adults, family carers and ageing research academics to meaningfully engage as partners in a research team guided by a partnership-focused framework
- Evaluate the impact of this research partnership on all members of the PPI panel.

## Study context

The study will be carried out in collaboration with the Ageing Research Centre (ARC) at the University Of Limerick, Ireland. The ARC comprises of an interdisciplinary group of researchers that aim to:

- Conduct excellent research that leads to improvement in the health, well-being and social inclusion of older people.
- Work across disciplinary boundaries to address research priorities that reflect the day to day realities of older people’s lives.
- Develop capacity in ageing research at UL and build collaborations with researchers, clinicians, industry partners, older people and their representative organisations.

The ARC membership includes academic researchers, postdoctoral researchers and PhD students. Four PhD students affiliated with the ARC are completing their doctoral studies on the “Right Care” Collaborative Doctoral Award (CDA) programme of research funded by the Health Research Board (HRB) in Ireland. The aim of the four aligned PhD projects is to advance the evidence base on appropriate, safe and cost-effective “Right Care” for older adults.

## Methods

The study will employ a qualitative participatory approach ^15, 30^ to data collection. To ensure rigour of the investigation, the consolidated criteria for reporting qualitative research (COREQ) ^31^ will be employed to guide data collection and analysis. Furthermore, the Guidance for Reporting Involvement of Patients and the Public (GRIPP2) ^32^ standardised reporting guidelines will be adopted. The GRIPP2 checklist was developed to improve the quality, consistency and reporting of patient and public engagement in research.

## Framework

A partnership-focused framework will guide the establishment of an older adult health services research stakeholder panel ^14^. This framework embeds INVOLVE principles and values ^33^. The authors (MC, KR, JS and RG) identified the participant “mix” that was required to set up this PPI group via discussion and consensus that older adults and family carers with health service utilisation experience were the most appropriate group to recruit. The academic researchers initiated the study, but it was recognised early on that PPI was crucial and involvement was planned for all subsequent stages. Furthermore, all CDA students are required to complete mandatory training in PPI as part of their doctoral projects.

## Recruitment

Identifying public contributors can be challenging ^34^; therefore, a broad and flexible recruitment strategy is planned. Recruitment will follow the principles of purposeful and snowball sampling ^15, 35^, drawing on existing contacts between the team, and collaborators and relevant agencies (Age Action Ireland, Family Carers Ireland). The first author (MC) will consult with older adults on the steering groups of Age Action Ireland and Family Carers Ireland to refine the recruitment strategy and recruitment flyer. We will use the following strategies for completing the PPI panel:

1. Public advertising in community locations, in community publications and community radio.
2. Advertising through patient organisation/community group/active retirement groups/Men’s sheds and family carer organisations as advised by the relevant agency steering groups (Age Action Ireland).
3. Established relationships and networks of ARC members will be explored to identify gatekeepers or community leaders in relevant organisations (Non government organisations public and community organisations) who could identify prospective participants.

After prospective participants express interest in joining the PPI panel, a researcher will schedule a meeting to discuss the information sheet and any queries the participant may have. The researcher will also assure participants that they will not, at any stage, be required to disclose any personal details of their own medical history or any other personal information. The researcher will refer to the INVOLVE guidelines on the knowledge, skills and experience required to participate in PPI when addressing participant queries ^36^. For example, participants should feel able to be reflective; confident and able to speak up in a group; be respectful of others in the group and have the confidence to question information and explanations supplied by others, who may be experts in their field, and have the ability to take an objective view, seeing issues from all perspectives. We plan to recruit 8–10 older adults, 4–6 family carers and 15 ARC members ^37^, acknowledging that not all ARC members will participate in all workshops/meetings.

## Panel involvement

The guidelines for involving older adults in research will be followed throughout the course of the study ^38^ in conjunction with INVOLVE principles for partnership frameworks . The following guidelines for involving older adults in research will be adhered to:

- Acknowledge research as a iterative process (the PPI panel of older adults will have input throughout various research projects at different stages of the research process)
- Provide training if required in order for older adults to participate in the research studies ^10^
- Clarify roles and determine levels of involvement to manage expectations
- Communicate effectively and be respectful of language difficulties
- Appreciate different expectations between academics, PPI panel of older adults and CDA students
- Acknowledge the concept of equal but different research partners ^27^.
- Specific needs of the PPI panel of older adults will be noted e.g hearing loss and the need for a large room with minimal noise and the use of a room with audio loop.

We will consider specific issues identified in the literature on how to involve older adults in research including:

- In any setting, family caregivers (defined to include family, friends, and other social support systems) play an important role in engaging and empowering older adults within the community
- Involvement opportunities need to be flexible (e.g., location, mode of interview, time, type)
- Incentivising involvement for researchers, patients and the public (financial and otherwise) may be necessary
- The education and training of citizens, health and social care providers, and researchers on involvement practices
- Patient-centered care approaches should consider the specific needs of individuals living with frailty including end-of-life care and advanced care planning
- Influencing policy can occur in many ways including participating at institutional, regional, provincial or national committees that relate to health and social care.
- Ensure representativeness and diversity while acknowledging that there is no single blueprint for involvement, as research involves working with a diversity of perspectives ^39^.

## Workshops/Meetings

A series of workshops involving the PPI panel of academic researchers from the ARC, older adults and family carers will be scheduled four times per year commencing in February 2020. The workshops will be approximately of 2 hours duration. The PPI group of older adults will have an active role at all stages of the research process for the ’Right Care’ CDA programme, ensuring that stakeholders are meaningfully involved at all relevant stages of the research process, particularly in identifying knowledge and action priorities for each of the ’Right Care’ doctoral projects, and interpreting and disseminating findings.

An initial meeting will consist of building a rapport with the members ^35^ and will be chaired by the authors (MC, KR, JS). The facilitators will set the context to the event by describing the aim of the meeting and providing a clear description of the Right Care programme. Consent will be obtained from all panel members, academics, older adults and family carers at the outset of the first meeting including consent for meetings to be recorded. If there are objections from members to recording we will try to achieve consensus through discussion. In the event that meetings are not recorded, detailed notes will be made during meetings with consent from participants. The principles of a World Café ^36^ and ground rules for participation will be discussed. Participants will be invited to discuss a number of issues relating to the Right Care CDA programme. During the four meetings over the course of a year, participants will take part in a variety of activities over a period of time to support the Right Care CDA students. A ratio of ARC members and CDA students will attend relating to their specific project to manage balancing of membership of the panel. Participants may be asked for their expertise for a number of projects including developing consent forms for recruitment in a trial; the development of lay summaries of the projects; assistance with the feasibility of the content of a pilot trial and the most appropriate method of dissemination of key research findings.

A number of interim reflexive meetings will be conducted throughout the year and records of interim reflective meetings will be collected. All correspondence, minutes of the meetings and other documents used during consultation activities will be logged. To address the second research aim of evaluating the impact of the research partnership on all members of the PPI panel we provisionally propose to record an activity log, maintain a record of all meetings and panel discussions. Individual interviews with all members of the research team will be conducted at key time points (after the initial meeting, 12 and 24 months post establishment of the panel). The final plan for evaluation will be negotiated and agreed with all members of the PPI panel.

A sample of a proposed participatory approach to engaging our PPI group as research partners, the World Café approach, is outlined below. The methodology is based upon the World Café’s seven integrated principles ^40^.

## The World Café Approach

The World Café principles and guidelines emphasise the importance of creating a hospitable environment (i.e. a café-style ambience) where individual and collective knowledge and ideas can be shared. There is no pressure to reach consensus, as diverse perspectives are encouraged and valued.

**Process:** Participants will be invited to discuss aspects of the proposed intervention. Tables will be covered with white paper ‘tablecloths’ and participants will be provided with pens and markers, facilitating participants to take notes on the paper tablecloth if they wish. At the end of the ‘table’ discussion, participants will rotate to another table as a group to discuss a different aspect of the proposed intervention. Participants will self-facilitate their own discussions, reinforcing the view that there is no hierarchy in the group. Self-recording will be used to allow less confident participants to draw or write their contribution. Facilitators at each table will ensure the write-up of notes resulting from each group discussion. Discussions at individual group tables will be recorded to capture insightful data.

**Co-analysis**: After all groups have rotated around all tables, knowledge from all the discussions will be shared with the full participant group. The café facilitators will facilitate the summary and co-analysis of the responses. Participants will then be provided with sticky, coloured paper dots and each participant will have the opportunity to vote, e.g. for their top three responses to each question.

## One-to-one interviews

All PPI panel members will be invited to complete a one-to-one semi-structured interview either over the phone or in person 12 and 24 months after the panel is formed. The panel members will be questioned as to their experiences of the research partnership. Specific research questions regarding each Right Care CDA projects will allow combining PPI with qualitative research ^35^. The interview will explore similar topics as investigated in the World Café groups with the addition of prompts and follow up questions. Interviews are anticipated to last between 40 and 60 minutes, will be audio-recorded electronically (with the written consent of the participant) and transcribed in full.

## Data analysis

Data transcription, analysis, interpretation and write-up will be carried out by the first author (MC) and the lead author (KR). Efforts will be made to ensure that both authors do not use leading language during data collection or shared personal experiences while maintaining a compassionate and positive attitude. The authors will strive to maintain a neutral and unbiased perspective during the analysis and interpretation of the data, facilitated by a process of “virtual double coding” where regular team discussions during the analysis, including meaningful participation of PPI members, allows coding and thematic categories to be critiqued and strengthened as the analysis progresses ^22^.

Transcribed one-to-one interviews recordings and transcribed World Café group notes will be entered into the qualitative data analysis software NVivo 12 Pro. A descriptive thematic approach to data analysis will be employed to provide a rich and detailed account of the data. All panel members will be invited to participate in collaborative data analysis, and we acknowledge data analysis as a collaborative and iterative process ^22^. An inductive approach to analysis will be undertaken linked to the research project aims. The six stage guide to thematic analysis described by Braun and Clark ^41^ will be adhered to: 1) familiarisation with the data through repeated reading; 2) identification of initial codes; 3) sorting of codes into potential themes; 4) review and discussion of themes by the two researchers to ensure internal homogeneity and external heterogeneity; 5) naming and definition of themes; 6) final analysis and generation of underlying story linking the themes. Additionally, there will be a member checking stage where the codes and themes will be discussed with representatives from the PPI panel of older adults. Through the process of returning and discussing results with PPI participants, an additional layer of interpretative value will be added ^42^.

## Ethical approval

PPI does not, in and of itself, require ethical approval. If, however, the PPI process is evaluated and the participants transcend their roles as co-researchers to become sources of data, then ethical approval is necessary ^43^. Our participatory research approach is at the interface between PPI and qualitative research and the principles do not account for ethical approval which might be required when there is such overlap. Therefore, we considered it necessary to obtain approval from a Research Ethics Committee to ensure that our approach is consistent with the Helsinki declaration and not over-burdening service users ^44^. The University of Limerick, Education and Health Sciences (EHS) Research Ethics Committee (2019_ 10_12_ EHS) approved the study (10 ^th^ October 2019). This study is funded by the Health Research Board (HRB) and the Health Research Institute (HRI), University of Limerick (UL) and will be conducted between November 2019 and October 2022.

## Discussion

Population ageing is an international priority with a urgent need for high quality multidisciplinary research to inform ageing policy ^38^. PPI is increasingly recognised as an integral part of the research process ^28^ with numerous positive impacts throughout the research process ^9, 11^. To ensure the relevance of research outcomes, it is essential that the views and experiences of older people are taken into account when designing research ^45^. The available literature on PPI involving older adults indicates that there are many benefits and challenges of engaging older adults in the research process, but challenges can be overcome. There is a need to build evidence that involvement has a positive impact on research processes ^25, 46^. This paper describes the methodological approach adopted to recruit and establish a PPI panel of older adults, family carers and academic researchers and our plans for describing the process and evaluating its impact on the research panel.

## Data availability

No data are associated with this article
