# Supplementary material for: A protocol for the establishment and evaluation of an older adult stakeholder panel for health services research
Source: HRB Open Res. 2020 Jul 20;3:1. Originally published 2020 Jan 14. [Version 2] doi: 10.12688/hrbopenres.12979.2 (PMC7374807; doi:10.12688/hrbopenres.12979.2)
Supplement: Peer review history

# Peer review: Reviewer response for version 2

**DOI:** 10.21956/hrbopenres.14210.r27694

**Published:** 2020-07-21

**Authors:** Éidín Ní Shé

**Affiliations:** 1 School of Nursing, Midwifery and Health Systems, C129, UCD Health Sciences Centre, University College Dublin, Dublin, Ireland

Thank you for the responses. This work will make a significant contribution to the literature around the best practice of undertaking PPI. I wish the team all the best and look forward to following the work.

Is the study design appropriate for the research question?

Partly

Is the rationale for, and objectives of, the study clearly described?

Yes

Are sufficient details of the methods provided to allow replication by others?

Partly

Are the datasets clearly presented in a useable and accessible format?

Yes

Reviewer Expertise:

Health Systems, Co-Design, Public and Patient Involvement.

I confirm that I have read this submission and believe that I have an appropriate level of expertise to confirm that it is of an acceptable scientific standard.

# Response

**Published:** 2020-07-21

**Authors:** Mairead Conneely

**Affiliations:** University of Limerick, Ireland

Many thanks for taking the time to review the revised manuscript and comments.

Kindest regards

Mairéad Conneely

# Peer review: Reviewer response for version 1

**DOI:** 10.21956/hrbopenres.14065.r27201

**Published:** 2020-03-27

**Authors:** Hanne Bruhn, Louisa Lawrie

**Affiliations:** 1 Health Services Research Unit, University of Aberdeen, Aberdeen, UK; 2 University of Aberdeen, Aberdeen, UK

**Summary**

 The study protocol by Conneely *et al.* (2020), proposes to establish a public and patient involvement (PPI) panel consisting of older adults, family carers, and ageing research academics to address the need to include the perspective of older adults in applied health research. The aim of the study is to describe setting up a PPI panel and to evaluate the impact of membership on all members. The PPI panel meetings will be conducted in the “world café” style and the authors will conduct semi structured interviews after 12 and 24 months. Thereafter, an inductive thematic analysis will be conducted on all available data including workshop and individual interview data.

**General comment**

 Although the study is about evaluating PPI impact and the authors describe the gold standard for involvement as having PPI onboard from the get-go, there is no PPI involvement in the current proposed evaluation study. It would be beneficial to include the authors’ considerations and justifications for this in the paper. This would fit in either the methods or discussion, depending on how the authors wish to present it. 

**Introduction**

 There appears to be a need in the research community to on one side evaluate the contribution public and patients make to research although they clearly are stakeholders, while on the other there is no such need as this is not done for other stakeholders such as the research fellow or statistician. It would be good if the authors could mention this duality somewhere in the paper. It could be early in the rationale for doing the study, or the Discussion in recognition of the different views.

 Given the increase in the healthy ageing population and the increasing number of older adults identifying as regular health service-users, it has become important to involve the older generation and provide them with an opportunity to shape decisions which may ultimately affect the healthcare that they receive. For this reason, the authors should be commended for this timely intervention. However, it may be more appropriate to highlight the need to involve older adults in a more positive light at the beginning of the article: rather than describing older adults as a burden for the healthcare system, it is an opportunity to increase representation of the ageing population. This would harmonise with the content outlined at the beginning of the discussion session, where the authors describe population ageing as an international priority and an older PPI panel as an integral part of the research process.

**Methods**

 The authors should be clearer on exactly *how *they will consider specific issues identified in the literature on engaging older adults. While it is suggested that they will attempt to address these issues, it would have been helpful to highlight how each issue will be addressed with the study design/methods outlined. For example, when conducting one-to-one interviews, the authors should indicate whether they will be flexible to adapting the location of the interviews to meet the older adults’ needs. It would also be helpful to highlight a justification for the timepoints of conducting these interviews (12 and 24 months). It may be worth considering an early 6 month data collection period to establish first impressions of the formation of the PPI. This would allow researchers to compare early vs. late attitudes, and subsequently identify any barriers and facilitators that may change overtime.

 The World Café Approach is an interesting method of collecting data. The authors should consider recording individual group tables to capture valuable data which may be discussed but not written on the white paper tablecloths. 

 Please provide either a justification or the considerations of the team when deciding on the number of people from different groups to be recruited for the panel. Although not all ageing researchers will attend every meeting there might be drop-outs etc in the older adult group or family carer group which might make balancing membership tricky. This information will also be of great help to researchers looking to this protocol for guidance on setting up a similar PPI panel.

 There is no record of any practical considerations for involvement of older adults e.g. if panel members withdraw or consideration of how to make involvement ‘easier’ for this population e.g. by providing documents in larger font, having meetings in a space where background noise will not be an issue (or even provide an audio induction loop). Any such provision is likely to help ‘recruitment’ and continued panel membership. The easiest way to address this would be to check with interested prospective panel members what their needs are. This might be a matter of course for the authors in which case a mention will be helpful to other researchers looking for guidance. In case of withdrawals – will members be replaced or how many members need to withdraw before replacements will be considered?

 It is a complex study and it would be good if the authors would help the reader more so they are better able to distinguish between the PPI panel evaluation study and the PPI panel activities while making clear that all PPI panel outputs contribute to the evaluation study. Particularly the section on ‘One-to-one interviews’ is currently difficult to follow and we think it would be helpful to give more descriptive details of the approach. The authors should also consider describing the approach used, just a short description, earlier in the methods, to help prepare readers.

 The authors use the terms ‘involvement’, ‘engagement’ and ‘participation’ seemingly interchangeably which can be confusing when the study is specifically about involvement. It would be good if the authors would review the use of the other terms (engagement and participation) for this reason. For example page 5, first paragraph – reference is made to ‘engagement’ throughout the points.

**Data analysis**

 On page 6, in the section on ‘Data analysis’ the authors state:

 “The authors will strive to maintain a neutral and unbiased perspective during the analysis and interpretation of the data.” This is ambitious and strictly speaking not possible. Some possible biases will have to be acknowledged e.g. research colleagues are panel members. One way of dealing with this would be to have someone independent of the study do the analysis or to have two people look at the data independently and then come to an agreement if there are discrepancies between them. There are likely to be other additional options which the authors might prefer.

**Data availability**

 In the section on data availability it would be good if the authors could clarify whether there is a plan for the data from the study to be made available for secondary analysis and how requests will be dealt with.

**Other small observations:**

- Page 4, paragraph on ‘Panel Involvement’, there is a space too much at the end of the following sentence:
  “The guidelines for involving older adults in research will be followed…”
- Page 5, first bullet point, the abbreviation “CDA” has not been defined previously.
- Page 6, 2 ^nd^ paragraph, the following sentence does not make sense:
  “Additionally, **there will be have **a member checking stage where the codes and themes will be discussed with representatives from the PPI panel of older adults.” 
- Page 6, Discussion paragraph, the following sentence is confusing:
  “Insufficient published examples of how to conduct effective PPI with older people exist.”

Is the study design appropriate for the research question?

Partly

Is the rationale for, and objectives of, the study clearly described?

Yes

Are sufficient details of the methods provided to allow replication by others?

Partly

Are the datasets clearly presented in a useable and accessible format?

Not applicable

Reviewer Expertise:

NA

We confirm that we have read this submission and believe that we have an appropriate level of expertise to confirm that it is of an acceptable scientific standard.

# Response

**Published:** 2020-07-12

**Authors:** Mairead Conneely

**Affiliations:** University of Limerick, Ireland

Many thanks to the reviewers for their useful feedback and comments. I have reflected on their feedback and revised the manuscript in line with their feedback.

**Point 1:**

Although the study is about evaluating PPI impact and the authors describe the gold standard for involvement as having PPI onboard from the get-go, there is no PPI involvement in the current proposed evaluation study. It would be beneficial to include the authors’ considerations and justifications for this in the paper. This would fit in either the methods or discussion, depending on how the authors wish to present it. 

**Response:**

Both reviewers raised this as an issue, and we appreciate the inherent problem with writing the protocol in advance of recruiting stakeholders. To address this in the protocol we have emphasised that the evaluation plan is provisional and will be discussed and negotiated with participants.  The research team are open to the planned evaluation changing however wanted to commence working with the group with ethical approval in place to allow meeting data to be captured. We have edited the Methods section to attend to the comments.

**Point 2:**

There appears to be a need in the research community to on one side evaluate the contribution public and patients make to research although they clearly are stakeholders, while on the other there is no such need as this is not done for other stakeholders such as the research fellow or statistician. It would be good if the authors could mention this duality somewhere in the paper. It could be early in the rationale for doing the study, or the Discussion in recognition of the different views.

**Response:**

Thank you for requesting clarity on this. While the study was initiated by the academic researchers, it was recognised early on that PPI was crucial and involvement was planned for all subsequent stages. I have edited the introduction to explain the rationale for doing this study as well as the Methids section (Heading: Framework). 

**Point 3:**

However, it may be more appropriate to highlight the need to involve older adults in a more positive light at the beginning of the article: rather than describing older adults as a burden for the healthcare system, it is an opportunity to increase representation of the ageing population. This would harmonise with the content outlined at the beginning of the discussion session, where the authors describe population ageing as an international priority and an older PPI panel as an integral part of the research process.

**Response:**

We value this feedback. It wasn’t our intent to communicate a view of older adults as a societal burden. We have reworded this paragraph.

**Point 4:**

The authors should be clearer on exactly *how *they will consider specific issues identified in the literature on engaging older adults. While it is suggested that they will attempt to address these issues, it would have been helpful to highlight how each issue will be addressed with the study design/methods outlined. For example, when conducting one-to-one interviews, the authors should indicate whether they will be flexible to adapting the location of the interviews to meet the older adults’ needs. It would also be helpful to highlight a justification for the timepoints of conducting these interviews (12 and 24 months). It may be worth considering an early 6 month data collection period to establish first impressions of the formation of the PPI. This would allow researchers to compare early vs. late attitudes, and subsequently identify any barriers and facilitators that may change overtime.

**Response:**

Thank you for requesting clarity. I have edited the Methods section here to highlight the flexibility of location/mode of interview.  Regarding time points the team agree that it is a great idea to evaluate early @ 6 months and plan/address issues as they come up. 

**Point 5:**

 The World Café Approach is an interesting method of collecting data. The authors should consider recording individual group tables to capture valuable data which may be discussed but not written on the white paper tablecloths. 

**Response:**

Thank you the authors have added this to the Methods of the World Cafe Approach

(Heading Process)

**Point 6**:

Please provide either a justification or the considerations of the team when deciding on the number of people from different groups to be recruited for the panel. Although not all ageing researchers will attend every meeting there might be drop-outs etc in the older adult group or family carer group which might make balancing membership tricky. This information will also be of great help to researchers looking to this protocol for guidance on setting up a similar PPI panel.

**Response:**

Thank you for this question. I plan to address this by keeping a certain ratio of ARC members or only certain CDA students will attend relating to their specific project. I have edited the Methods (Heading: Workshops/Meetings) to reflect this change.

**Point 7:**

There is no record of any practical considerations for involvement of older adults e.g. if panel members withdraw or consideration of how to make involvement ‘easier’ for this population e.g. by providing documents in larger font, having meetings in a space where background noise will not be an issue (or even provide an audio induction loop). Any such provision is likely to help ‘recruitment’ and continued panel membership. The easiest way to address this would be to check with interested prospective panel members what their needs are. This might be a matter of course for the authors in which case a mention will be helpful to other researchers looking for guidance. In case of withdrawals – will members be replaced or how many members need to withdraw before replacements will be considered?

**Response:**

Thank you for this comment. This would follow who we recruit and what their specific needs are. Hearing loss etc. The location can be flexible to allow a large room with minimal noise and use of a room with an audio loop-again depending on what the members require.  I have added this point to Methods section (Panel Involvement).

**Point 8:**

It is a complex study and it would be good if the authors would help the reader more so they are better able to distinguish between the PPI panel evaluation study and the PPI panel activities while making clear that all PPI panel outputs contribute to the evaluation study. Particularly the section on ‘One-to-one interviews’ is currently difficult to follow and we think it would be helpful to give more descriptive details of the approach. The authors should also consider describing the approach used, just a short description, earlier in the methods, to help prepare readers.

**Response:**

Thank you for commenting on the complexity of the study. I have edited the early part of the Methods section to clarify the confusion. To address the second research aim of evaluating the impact of the research partnership on all members of the PPI panel we provisionally propose to record an activity log and maintain a record of all meetings, record panel discussions and conduct individual interviews with all members of the research team at key time points (after the initial meeting, 12 and 24 months post establishment of the panel). The final plan for evaluation will be negotiated and agreed with all members of the PPI panel.

**Point 9:**

The authors use the terms ‘involvement’, ‘engagement’ and ‘participation’ seemingly interchangeably which can be confusing when the study is specifically about involvement. It would be good if the authors would review the use of the other terms (engagement and participation) for this reason. For example page 5, first paragraph – reference is made to ‘engagement’ throughout the points.

**Response:**

Thank you for requesting the use of one term. I had used the terms to reflect the terms used in the specific reference we cited. I have now used the term INVOLVEMENT throughout for consistency.

**Point 10:**

Data Analysis

“The authors will strive to maintain a neutral and unbiased perspective during the analysis and interpretation of the data.” This is ambitious and strictly speaking not possible. Some possible biases will have to be acknowledged e.g. research colleagues are panel members. One way of dealing with this would be to have someone independent of the study do the analysis or to have two people look at the data independently and then come to an agreement if there are discrepancies between them. There are likely to be other additional options which the authors might prefer.

**Response:**

Thank you for this comment. I have reworded the section to "The authors will strive to maintain a neutral and unbiased perspective during the analysis and interpretation of the data, facilitated by a process of ‘virtual double coding’ where regular team discussions during the analysis, including meaningful participation of PPI members, allows coding and thematic categories to be critiqued and strengthened as the analysis progresses."

**Point 11:**

Page 4, paragraph on ‘Panel Involvement’, there is a space too much at the end of the following sentence:

“The guidelines for involving older adults in research will be followed…”

**Response: **Thank you, this has been edited.

Page 5, first bullet point, the abbreviation “CDA” has not been defined previously.

**Response: **Thank you, this has been introduced under Methods (Heading: Study Context).

Page 6, 2nd paragraph, the following sentence does not make sense:

“Additionally, **there will be have **a member checking stage where the codes and themes will be discussed with representatives from the PPI panel of older adults.” 

**Response: **Thank you, this has been edited.

Page 6, Discussion paragraph, the following sentence is confusing:

“Insufficient published examples of how to conduct effective PPI with older people exist.”

**Response: **Thank you, this has been edited to **"**There is a need to build evidence that involvement has a positive impact on research processes with references".

# Peer review: Reviewer response for version 1

**DOI:** 10.21956/hrbopenres.14065.r27084

**Published:** 2020-01-30

**Authors:** Éidín Ní Shé

**Affiliations:** 1 School of Nursing, Midwifery and Health Systems, C129, UCD Health Sciences Centre, University College Dublin, Dublin, Ireland

This is a well written and very welcome protocol outlining an evaluation proposal to capture learnings of the establishment of a PPI panel involving older people and family carers.

 My only reservation is to request clarity from the team on the following:

- What involvement has occurred with older people and family carers on the establishment of the panel and the proposed evaluation as outlined?
- What will the team do if members recruited do not agree with the evaluation as proposed?
- Who holds the power in understanding and interpreting the data? How will members of the PPI panel be supported if they want to be involved in analysis?
- What strategies have the team devised if members of the PPI panel object to having their sessions recorded?
- How will the knowledge created be shared with PPI panel members?

Minor comment:

 First paragraph: Overall this opening paragraph from my readings seems to place blame on our older population for being a burden on the health services: “As older adults bear the greatest burden of disease, they account for the greatest proportion of healthcare use” - where is the evidence for this?

 Our ageing population it can be argued is the result of advances in our health system a point that should be celebrated. This shift points to the need to rethink the reconfiguration of our services and enabling older peoples involvement in what they want should be at the core of this shift. I would suggest a reframing of this opening paragraph.

Is the study design appropriate for the research question?

Partly

Is the rationale for, and objectives of, the study clearly described?

Yes

Are sufficient details of the methods provided to allow replication by others?

Partly

Are the datasets clearly presented in a useable and accessible format?

Yes

Reviewer Expertise:

Health Systems, Co-Design, Public and Patient Involvement.

I confirm that I have read this submission and believe that I have an appropriate level of expertise to confirm that it is of an acceptable scientific standard, however I have significant reservations, as outlined above.

# Response

**Published:** 2020-07-12

**Authors:** Mairead Conneely

**Affiliations:** University of Limerick, Ireland

Many thanks for taking the time to review this protocol. I apologise for the delay with the response. 

**Point 1:**

My only reservation is to request clarity from the team on the following:

What involvement has occurred with older people and family carers on the establishment of the panel and the proposed evaluation as outlined? 

What will the team do if members recruited do not agree with the evaluation as proposed?

**Response**: 

Both reviewers raised this as an issue, and we appreciate the inherent problem with writing the protocol in advance of recruiting stakeholders. To address this in the protocol we have emphasised that the evaluation plan is provisional and will be discussed and negotiated with participants.  The research team are open to the planned evaluation changing however wanted to commence working with the group with ethical approval in place to allow meeting data to be captured. The Methods section now reflects these changes.

**Point 2:**

Who holds the power in understanding and interpreting the data? How will members of the PPI panel be supported if they want to be involved in analysis?

**Response:**

Thank you for this comment. In the Methods Section (Heading: Data Analysis), I  have clarified that all panel members will be invited to participate in collaborative analysis of the data. facilitated by a process of “virtual double coding” where regular team discussions during the analysis, including meaningful participation of PPI members, allows coding and thematic categories to be critiqued and strengthened as the analysis progresses.

**Point 3:** 

What strategies have the team devised if members of the PPI panel object to having their sessions recorded?

**Response: **Thank you for requesting clarity on this. Consent will be obtained from all panel members , academic and older people and family carers, at the outset of the first meeting and includes consent for meetings to be recorded. If there are objections to recording meetings from members we will discuss and  if consensus is that sessions should not be recorded we will discuss if detailed notes can be recorded.This has now been detailed in the Methods section (Heading: Workshops/Meetings).

**Point 4:**

How will the knowledge created be shared with PPI panel members?

**Response:**

Thank you for this question. We have reflected on this and clarified in the Methods section (Data Analysis), Through returning and discussing results with PPI participants, thus adding an additional layer of interpretive value as outlined in MacCauley et al 2007. All panel members will be invited to participate in collaborative data analysis, and we acknowledge data analysis as a collaborative and iterative process. 

**Point 5: Minor comment:**

First paragraph: Overall this opening paragraph from my readings seems to place blame on our older population for being a burden on the health services: “As older adults bear the greatest burden of disease, they account for the greatest proportion of healthcare use” - where is the evidence for this?

Our ageing population it can be argued is the result of advances in our health system a point that should be celebrated. This shift points to the need to rethink the reconfiguration of our services and enabling older peoples involvement in what they want should be at the core of this shift. I would suggest a reframing of this opening paragraph.

**Response:**

We value this feedback. It wasn’t our intent to communicate a view of older adults as a societal burden. We have reworded this paragraph

****
